# Blood biomarkers for Alzheimer’s disease

**DOI:** 10.1186/s13073-014-0065-7

**Published:** 2014-08-31

**Authors:** Simon Lovestone

**Affiliations:** Department of Psychiatry, University of Oxford, Warneford Hospital, Oxford, OX3 7JX UK

## Abstract

Simon Lovestone discusses recent progress in the development of molecular biomarkers for the diagnosis and prognosis of Alzheimer’s disease.

## Introduction

**Figure 1:**
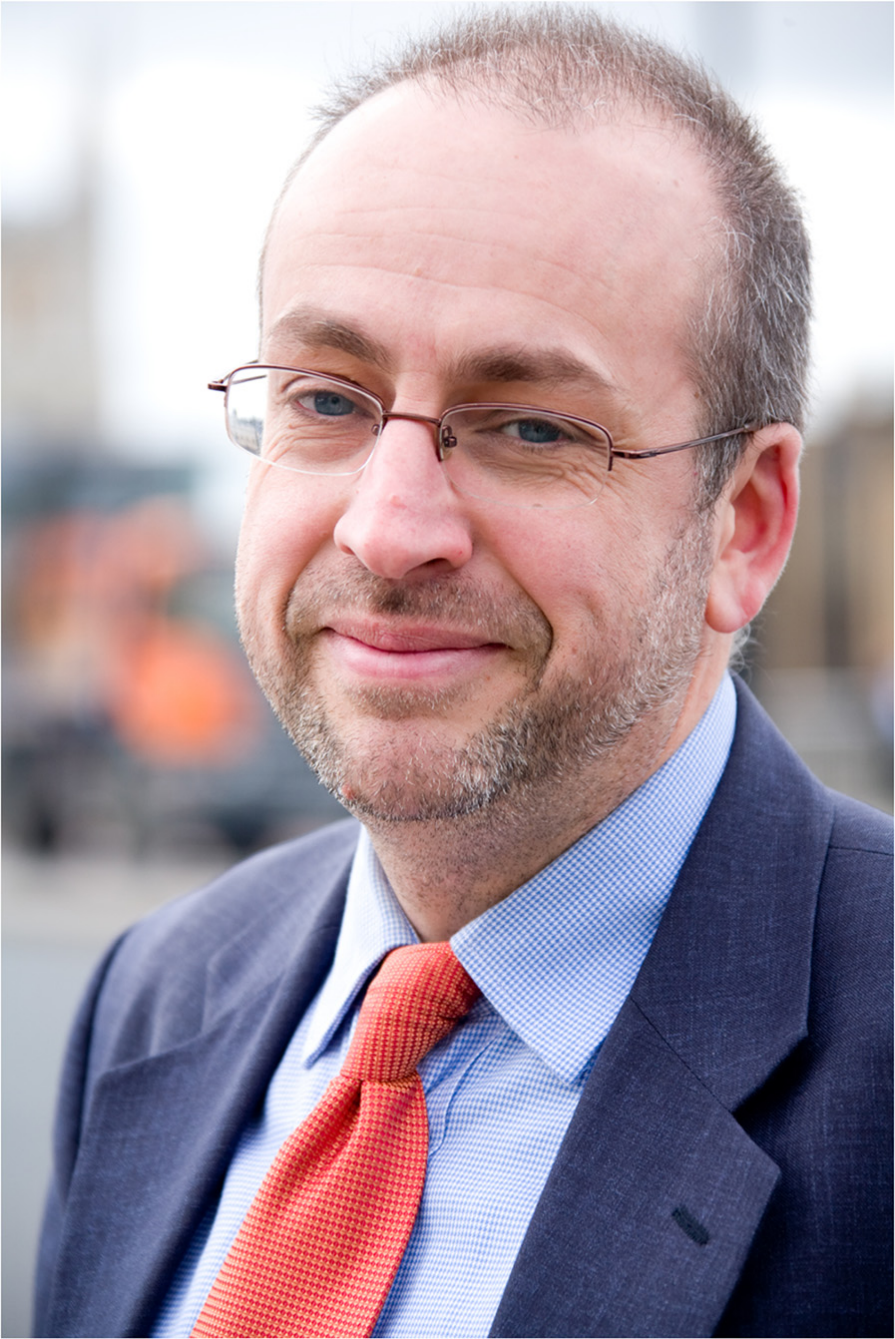
**Simon Lovestone.**

Simon Lovestone (Figure 1) is Professor of Translational Neuroscience at the University of Oxford and Lead for the NIHR Translational Research Collaboration in Dementia, a network of experimental medicine centers in the UK. He is a leading name in the research field of blood biomarkers for Alzheimer’s disease. In this Q&A, he shares with us his vision on the state of the art of the field and on what will be required in the future.

## What is the standard for diagnosis of Alzheimer’s disease currently?

The standard today is to diagnose dementia according to clinical criteria, for the most part using defined operational criteria, and with, for example, imaging used primarily to exclude other diseases. Diagnosis of Alzheimer’s disease (AD) and other dementias is actually reasonably effective and accurate, and this has been demonstrated in several studies, most of which concur on this point [1].

In fact, some years ago, we examined how accurate the diagnostic process was, when performed by trained non-medical researchers, using a standardized assessment process. When tested against post-mortem follow-up diagnosis, using such an assessment process, combined with a computerized algorithm, had a positive predictive value of at least 95% [2]. However, there are three caveats to this. First, AD is by far and away the most common of the dementias and so, in any cohort, the positive predictive value will be high for almost any diagnostic process. Second, all diagnostic processes have an underlying assumption that dementias are categorical disorders (Alzheimer’s, vascular dementias, fronto-temporal dementias, and so on) whereas, in fact, it would be more accurate to think of them as syndromes emerging from a continuum or spectrum of overlapping pathologies. Third, all these comments about diagnostic accuracy only apply when considering established dementia; the real difficulty is in identifying prodromal or even preclinical disease accurately. It is known that the disease process starts a decade or more before the full dementia syndrome is established. Especially when we want to do clinical trials earlier, identifying people in these pre-dementia states is really important but, today, really difficult.

## Is the current approach used for diagnosis of Alzheimer’s disease desirable?

Clearly this diagnostic approach - diagnosing disease up to 10 years after onset and only when the symptoms are so severe as to be obvious, and making categorical diagnoses when we know there is usually a mixture or spectrum of pathologies - is not desirable and is now increasingly outdated. In fact, it is worse than that. Not only is the diagnostic approach no longer adequate for established dementia but, also, doctors are increasingly being approached by people in the pre-dementia stages. Here, the usual practice, at least in the UK, is to perform an assessment of cognition and, if the patient does not have full dementia, to advise a reassessment after six months or a year. This period of waiting and uncertainty should be - and patients and their carers clearly tell me is - stressful and worrying.

## What recent advances have there been to identify biomarkers for Alzheimer’s disease?

Considerable progress has been made in the identification of biomarkers for AD using molecular markers in cerebrospinal fluid (CSF) and molecular imaging. Many groups have contributed to this, but the group of Kaj Blennow and his colleague Henrik Zetterburg have really led the field in establishing markers of pathology, including the levels of amyloid-beta, which decrease in CSF in AD, and microtubule-associated protein tau and phosho-tau levels, which increase in CSF in this condition [3].

More work is needed to resolve exactly how these markers change over time, their predictive value and also the current difficulty in reconciling analytical issues that give variable results in different laboratories. Nonetheless, these markers are now in clinical practice in many parts of the world - certainly in Scandinavia, increasingly in the USA, and also in many countries in Europe. Molecular imaging is also increasingly used, especially in clinical trials. The use of positron emission tomography (PET) ligands for amyloid is well established, and Tau ligands are on their way.

The Alzheimer’s Disease Neuroimaging Initiative has done much to establish the diagnostic and predictive value of amyloid PET imaging as well as CSF molecular markers and structural magnetic resonance imaging (MRI) [4].

In early June 2014, data from the Alzheimer’s Disease Neuroimaging Initiative and from the European biomarkers study - AddNeuroMed [5] - were released together as part of the Sage Bionetworks Dream Challenge to find biomarkers for AD – an example of crowdsourcing solutions to the diagnostic dilemma using Big Data.

## What are the prospects for identifying blood-derived biomarkers for early diagnosis or prognosis?

My group has long focused on trying to find biomarkers in blood [6,7]. A decade or so ago, when we started, it seemed unlikely that such markers would be found. Indeed, we set off to prove the null hypothesis. However, we soon found that, by using proteomics, we could identify a signal - a set of spots on two-dimensional gel electrophoresis and identified by mass spectrometry - and set out to try and pin this signal down to some molecules for use as biomarkers. We have used proteomics, genomics, metabolomics and informatics to try to do this and used these alone, as well as together and with imaging markers. Many other groups have joined the search for blood-based biomarkers, reporting some really interesting results that, like our data, will need replication.

Along the way, we have learnt three main lessons, but still have a long way to go. First, we have come to appreciate that samples and data matter. We need many samples, well curated and with as many data from other techniques measuring the same analytes as possible. In fact, we think that no one group can hope to accrue all the samples necessary for such studies and that the future lies with data-aggregation in collaborations such as the IMI-European Medical Information Framework and the UK Dementias Platform. Second, we have learnt it makes no sense to do the conventional case-control study when we are trying to find markers for occult or prodromal disease. For this reason, my lab has moved to an endophenotypic approach. This predicates discovery on another marker of disease - so, rather than comparing disease cases with healthy controls, we compare or correlate markers in blood with - variously - rate of decline, structural MRI data, PET amyloid data or CSF markers of disease. The point is that only some healthy controls are free from disease and many have the same amount of disease as the diseased cases. In fact, all of the people we want to identify with our markers are actually in the control group. Therefore, this makes no sense as a study design. Instead, by either disregarding disease category, or including only people in one category, such as mild AD, we can identify markers that correlate with, and perhaps are surrogates for, actual pathology. Third, we have found that most ‘omics technologies remain in their infancy and that no technical platform is sufficiently superior to others to justify the enthusiasm sometimes expended on them by their supporters. Having spent the past decade looking for blood biomarkers, I’m convinced that it isn’t the discussion around proteomics versus metabolomics, or mass spectrometry versus antibody capture that matters, but finding the best way to utilize elements of all these approaches in combination. A combinatorial approach seems the only sensible way forward to me.

Given all this, I think that the evidence suggests that the prospects for identifying blood-derived markers for early diagnosis and prognosis are very good [8]. Despite the somewhat superficial comments one sometimes hears about ‘fishing expeditions’ and ‘non-replication’, in fact we are learning some substantial biology and mechanisms from these studies, and the replication of some markers across multiple studies is actually rather impressive [9].

## Could blood biomarkers also be used to monitor the progression of Alzheimer’s disease?

This is less certain. There is very little evidence today that markers can be used to track disease. However, we have started a study as part of the UK Dementia Platform, called the Deep and Frequent Phenotyping study, where the ‘deep phenotyping’ refers to hugely multimodal assessments, including PET, MRI, electrophysiology, a range of clinical and cognitive measures and molecular measures in CSF and blood, and the ‘frequent phenotyping’ refers to our plans to do much of this multimodal phenotyping repeatedly, precisely to identify the marker set most able to track disease.

## Which type of biomarkers do you think would be more likely to translate into the clinic?

Whether, when and how blood-based biomarkers being developed in research get translated into the clinic depends somewhat on the data, and somewhat - possibly a greater somewhat - on health-service-related issues. These include the ease of requesting and performing the biomarker test, the cost and also the value to the health services. So, all the biomarkers under consideration become considerably more valuable, and possibly even indispensable, if a therapy becomes available that is partnered with a marker. The likelihood of translation is therefore a combination of acceptability to patients - where blood wins hands down - to accuracy and utility - where today CSF measures, possibly, have the edge to ease of use and interpretation - while, from a physician’s perspective, PET might be a front-runner, where available. In time, the decision-making is likely to become rationalized around the evidence, which today is far from complete.

## How far are we from using blood-based biomarkers in the clinic?

Even now, CSF measures are used in clinical practice in many parts of the world. PET imaging is likely to be used in some clinical contexts quite soon. Whether blood-based biomarkers ever get used in the clinic is an open question. Ten years ago, I would have predicted that they would never reach the accuracy levels required for clinical utility. Today, as we have just published data showing an accuracy in prediction of progression from preclinical syndromes to dementia of around 80%, I am not so sure [10]. It rather looks to me as though blood-based biomarkers could be heading to the clinic, perhaps as part of a suite of diagnostics. Very many more studies will have to be performed to ascertain test performance if such tests are to be used in a clinical context and not just in the research context as we planned. How long this will take and whether the results in larger and more-community-based populations will maintain test outcomes is hard to say. Prediction is always difficult - especially about the future!

## Abbreviations

AD: Alzheimer’s disease
CSF: Cerebrospinal fluid
NIHR: National Institute for Health Research
MRI: Magnetic resonance imaging
PET: Positron emission tomography
